# STK24 modulates excitatory synaptic transmission in epileptic hippocampal neurons

**DOI:** 10.1111/cns.13391

**Published:** 2020-05-21

**Authors:** Juan Yang, Qian Jiang, Xinyuan Yu, Tao Xu, You Wang, Jing Deng, Yong Liu, Yangmei Chen

**Affiliations:** ^1^ Department of Neurology the Second Affiliated Hospital of Chongqing Medical University Chongqing China

**Keywords:** epilepsy, neurons, NMDA receptors, STK24, synaptic transmission

## Abstract

**Introduction:**

A large amount of literature has indicated that excitatory synaptic transmission plays a crucial role in epilepsy, but the detailed pathogenesis still needs to be clarified.

**Methods:**

In the present study, we used samples from patients with temporal lobe epilepsy, pentylenetetrazole‐kindled mice, and Mg^2+^‐free‐induced epileptic cultured hippocampal neurons to detect the expression pattern of STK24. Then, the whole‐cell recording was carried out after STK24 overexpression in the Mg^2+^‐free‐induced epileptic cultured hippocampal neurons. In addition, coimmunoprecipitation was performed to detect the association between endogenous STK24 and main subunits of NMDARs and AMPARs in the hippocampus of PTZ‐kindled mice.

**Results:**

Here, we reported that STK24 was specifically located in epileptic neurons of human and pentylenetetrazole‐kindled mice. Meanwhile, the expression of STK24 was significantly down‐regulated in these samples which are mentioned above. Besides, we found that the amplitude of miniature excitatory postsynaptic currents was increased in STK24 overexpressed epileptic hippocampal cultured neurons, which means the excitatory synaptic transmission was changed. Moreover, the coimmunoprecipitation, which further supported the previous experiment, indicated an association between STK24 and the subunits of the NMDA receptor.

**Conclusion:**

These findings expand our understanding of how STK24 involved in the excitatory synaptic transmission in epilepsy and lay a foundation for exploring the possibility of STK24 as a drug target.

## 1 INTRODUCTION

Epilepsy is a chronic brain disease with the repeated, abnormal, and high‐synchronization discharge of neurons. About 1%‐2% of the population, nearly 65 million people, were affected by epilepsy around the world.^1^, ^2^ Although drug therapy is effective for a majority of patients, there are still up to 20%‐30% of patients are refractory and suffer from recurrent seizures.^3^

Up to date, the pathogenesis of epilepsy remains unclear and needs to be further clarified. The imbalance of excitation and inhibition of the neural circuit is considered to be one of the most important pathogenesis of epilepsy.^4^ Dendritic spines, which have mainly involved in the afferent of excitatory signals, are critical for the formation of functional neural circuits.^5^

Serine/threonine kinase 24 (STK24), which belongs to the sterile 20 kinase family, also known as mammalian sterile 20‐like kinase 3 (MST3), is widely expressed in many tissues including the brain.^6^, ^7^ STK24 was reported to facilitate dendritic spine development and maintain the structure of excitatory synapse.^8^ Further, STK24 was also involved in changing the spontaneous excitatory postsynaptic currents of the mouse cortical pyramidal neurons in upper layers II‐III, which indicates its possible role in regulating cortical excitability.^9^ However, it has not been studied whether STK24 modulates the excitatory synaptic transmission in epilepsy.

Here, we investigated the location and expression pattern of STK24 in epilepsy and observed the change of electrophysiology in the Mg^2+^‐free‐induced epileptic hippocampal neuronal culture model after overexpressing STK24. Finally, we determined the possible interaction between STK24 and several main glutamate receptors in the pentylenetetrazole (PTZ)‐kindled mouse model.

## 2 METHODS AND MATERIALS

### 2.1 Human samples

This study protocol was approved by the Ethics Committee of the Second Affiliated Hospital of Chongqing Medical University and complied with the Declaration of Helsinki and the ethical principles and guidelines of the National Institutes of Health. Patients were diagnosed with retractable TLE in accordance with the criteria proposed by the International League Against Epilepsy (ILAE).^10^ Control brain tissues were acquired from patients who underwent brain surgery owing to severe head trauma. All control patients had no history of epilepsy, seizures, or any other central nervous system disease and did not take any antiepileptic therapy before head trauma. Temporal cortical tissue samples (14 from refractory TLE patients, 10 from patients with severe head trauma) were obtained from the Xinqiao Hospital of Third Military Medical University and the First Affiliated Hospital of Chongqing Medical University, and the clinical data of these patients were reported in our previous research.^11^, ^12^ For the use of clinical data and brain tissue, informed consent was signed by the patients or their guardians.

### 2.2 PTZ‐kindled mouse model

The animal experiments in our research were approved by the ethics committee of Chongqing Medical University. All animal studies were conducted abiding by the National Institutes of Health Guide for the Care and Use of Laboratory Animals and the rules of the Animal Ethical Committee of Chongqing Medical University. Healthy male C57BL/6 mice (20‐25 g) were provided by the Experimental Animal Center of Chongqing Medical University. All of the mice were kept in a specific pathogen‐free animal facility under standard conditions at 21‐22°C with 50‐60% humidity in a 12 h/12 h light/dark cycle and were freely eating and drinking. Every effort had been made to minimize the suffering of the animals and their quantity.

The mice in the PTZ‐kindled model were intraperitoneally injected with a daily subconvulsive dose of PTZ (35 mg/kg, Sigma‐Aldrich, USA) between 9:00 AM and 11:00 AM.^13^, ^14^ The rest mice were assigned as controls and were injected with the corresponding dose of saline solution. The intensity of seizure was evaluated on the basis of Racine's scale evaluation^13^: 1: mouse and facial movements; 2: head nodding; 3: forelimb clonus; 4: rearing; and 5: rearing and falling. Mice were deemed fully kindled when exhibiting seizure attacks (score 4 or 5) after each PTZ injection for at least three consecutive days. The mice were anaesthetized with pentobarbital (6 mg/100 g) before sacrificed.

### 2.3 Neuronal culture preparation, DNA constructs, and transfection

Cultures of hippocampal neurons were prepared from postnatal day 0 C57BL/6J pups of either sex as mentioned.^14^, ^15^ Neurons were plated in DMEM containing 10% fetal bovine serum (FBS) (Scitecher, French), 1% penicillin‐streptomycin (P/S) (Gibco, USA), and 1% l‐glutamine (Gbico, Brazil). Four to six hours later, the culture medium was exchanged completely with the neurobasal medium containing 2% B27 supplement (Gbico, USA), 1% P/S, and 1% l‐glutamine. Thereafter, half of the serum‐free medium was exchanged with an equivalent volume of fresh serum‐free medium every three days. Neurons were plated in 6‐well plates with coverglasses at a density of 2 million cells/well for electrophysiology and 5 million cells/6 cm plates for Western blot analysis.

Flag‐tagged mouse STK24 plasmids with pcDNA mammalian expression vector were constructed and purchased from youBio, China. DIV (day in vitro) 10, neurons were transfected with Venus and Flag‐tagged empty vector pcDNA3.1 or Venus together with STK24 using a calcium phosphate method, respectively.^16^ The amount of total cDNA was balanced by adding pcDNA3.1 in control. During whole‐cell recordings, Venus was observed as an indicator of cell selection and the mEPSCs and mIPSCs were recorded to measure the postsynaptic currents.

### 2.4 Mg^2+^‐free‐induced spontaneous recurrent epileptiform discharge model of hippocampal neurons (SREDs)

Mg^2+^‐free‐treated neuronal model, which can simulate spontaneous recurrent epileptiform discharges, was set up to research epilepsy according to the previous study.^17^ At DIV10 (for Western blot) or 16 (for whole‐cell recordings), the serum‐free medium was exchanged with Mg^2+^‐free medium (in mM) (145 NaCl, 2.5 KCl, 10 HEPES, 2 CaCl2, 10 glucose, and 0.002 glycine, pH 7.2‐7.4, 280‐320 mOsm) or nonmagnesium‐free medium (non‐MGF, supplemented with 1 mM MgCl_2_) for three hours. Three hours later, the Mg^2+^‐free medium or non‐Mg^2+^‐free medium was discarded and the serum‐free culture medium was added in. Within 12‐24 hours, normal Mg2+ concentration in the culture has been restored and SREDs are typically observed and continue throughout the cell culture period. Then, neurons were harvested for Western blot (DIV 10) and whole‐cell patch‐clamp recordings (DIV 16).

### 2.5 Protein extraction and Western blot

Brain tissues of human and mouse were homogenized to extract total proteins via total protein extraction kit (Sangon, China). The concentration of protein was calculated via the BCA Protein Concentration Assay Kit (Dingguo, China). After denaturing by metal bath, protein (20‐50 µg) was loaded in each lane, separated by 10% SDS‐PAGE, and transferred to a 0.45 µm PVDF membrane (GE, USA). The PVDF membranes were blocked with 5% skimmed milk and then incubated with the following primary antibodies overnight at 4°C: MST3 (rabbit, monoclonal; 1:2000; ab51137; Abcam, USA), STK24 (rabbit, monoclonal; 1:1500; DF4463; Affinity, USA), GLUR1 (rabbit, polyclonal; 1:1000; abs136161; Absin, China), GLUR2 antibody (rabbit, monoclonal; 1:1000; CY7063; Abways, China), NMDAR1 (rabbit, polyclonal; 1:1000; AF6406; affinity, USA), NMDAR2A (rabbit, polyclonal; 1:500; 19953‐1‐AP; Proteintech, China), NMDAR2B (rabbit, monoclonal; 1:1000; 14544S; CST, USA), GABA_A_R α1 (rabbit, polyclonal; 1:500; 12410‐1‐AP; Proteintech, China), GABA_A_R γ2 (rabbit, polyclonal; 1:1000; 14104‐1‐AP; Proteintech, China), GABA_A_R β2/3 (rabbit, polyclonal; 1:500; bs‐12066R; BIOSS, China), and GAPDH (rabbit, polyclonal; 1:4000‐1:5000; 10491‐1‐AP; Proteintech, China).

On the second day, the PVDF membranes incubated with corresponding secondary antibodies at room temperature for 1 hour: HRP‐conjugated goat anti‐rabbit IgG (1:4000; SA00001‐2; Proteintech, China) and HRP‐Mouse Anti‐Rabbit IgG Light Chain Specific (1:5000; SA00001‐7L; Proteintech, China). Subsequently, the membranes were visualized via ECL substrate (P0018FS, Beyotime, China) and were imaged on Fusion imaging system (Vilber Lourmat, France). Optical density (OD) was acquired by quantity one and was normalized to the loading controls separately. Experiments were performed 3‐4 times independently under the same conditions. Uncropped images of Western blots are shown in Figure S1.

### 2.6 Immunofluorescence labeling and Confocal Microscopy

Immunofluorescence labeling was performed using previously established procedures.^18^, ^19^ The frozen sections were incubated with a mixture of the following primary antibodies overnight at 4°C: STK24 (rabbit, polyclonal; 1:50, bs‐7599R; BIOSS, China), MAP‐2 (guinea pig, polyclonal; 1:200, 188004; SYSY, Germany), and GFAP (mouse, monoclonal; 1:100, 60190‐1‐Ig; Proteintech, China).

On the next day, the sections were incubated with a mixture of corresponding secondary antibodies at room temperature for 1 hour: Alexa Fluor 488‐conjugated donkey anti‐mouse IgG (1:50; 115‐545‐003; Jackson ImmunoResearch, USA), Alexa Fluor 405‐conjugated donkey anti‐guinea pig IgG (1:50; 106‐475‐003; Jackson ImmunoResearch, USA), and Alexa Fluor 594‐conjugated goat anti‐rabbit IgG (1:50; 111‐585‐003; Jackson ImmunoResearch, USA) shielded from light for 60 minutes at 37°C. Images were captured by a confocal laser scanning microscope (A1 + R, Nikon, Japan).

### 2.7 Coimmunoprecipitation

Coimmunoprecipitation was conducted in the light of the manufacturer's manual of protein A/G magnetic beads (HY‐K0202, MedChem Express, USA). In short, 40 μl of protein A/G magnetic beads was added into an eppendorf tube and washed with 400 ul binding/wash buffer four times. Next, the beads were incubated with 3‐4 μg primary antibody as previously mentioned. Rabbit monoclonal IgG (ab172730, Abcam, USA) was also used as a negative control. Subsequently, the supernatant was discarded and the magnetic beads were washed with binding/wash buffer. Then, the magnetic beads were incubated with protein lysates which obtained from hippocampi of PTZ‐kindled mice. After washing, the supernatant was removed, and 1× SDS loading buffer was added. After denaturing by metal bath, the supernatants were collected for Western blot. Uncropped images of Western blots after coimmunoprecipitation are shown in Figure S1.

### 2.8 Whole‐cell recordings

The whole‐cell recording was carried out using previously established procedures.^17^ Briefly, DIV16‐17, a coverslip of neurons, was transferred to a chamber with an extracellular solution on the inverted microscope stage (IX51, Olympus, Japan). In the current‐clamp mode, action potential (AP) was recorded at the resting membrane potential. When APs were measured, the extracellular Mg^2+^‐free ACSF (in mM) (140 NaCl 140, 5 KCl, 10 HEPES, 1.8 CaCl2, 10 D‐Glucose) or non‐Mg^2+^‐free ACSF (supplemented with 1 mM MgCl2）was continuously perfused. The pipettes (3‐5 MΩ) were filled with an intracellular solution (in mM) (110 KAsp, 10 EGTA, 10 HEPES, 30 KCl, 5 Na‐ATP, 1 CaCl_2_, 2 MgCl_2_, and 10 TEACl, pH 7.3, 280‐300 mOsm). Also, mEPSCs were recorded. The glass pipettes were filled with the correspondent intracellular solution (in mM) (17.5 CsCl, 10 HEPES, 4 ATP, 0.5 EGTA,132.5 Cs‐gluconate, and 5 QX‐314, pH 7.2); meanwhile, the chamber was perfused with extracellular Mg^2+^‐free ACSF containing 10 μM bicuculline (to block GABA_A_ receptor‐mediated inhibitory synaptic currents) and 1 μM tetrodotoxin (to block action potentials) at the holding potential −70 mV. Besides, mIPSCs were similarly recorded but in the presence of 20 μM DNQX (Sigma‐Aldrich, USA) (to block selective non‐NMDA receptors), 50 μM AP5 (Sigma‐Aldrich, USA) (to block NMDA receptors), and 1 μM TTX. Whole‐cell recordings were conducted via a Multi‐clamp 700B amplifier (Axon, USA), Digidata1440A, pCLAMP10.0.3.1.

### 2.9 Data analysis

Shapiro‐Wilk test was used to verify the normal distribution, and Levene's test was used to analyze the homogeneity of variance. Fisher's exact test was used to compare the difference in sex distribution between TLE patients and control patients. For the two independent samples with normal distribution, if the variances are homogeneous, unpaired Student's *t* test is adopted to compare the difference between two groups; otherwise, Welch's *t* test is adopted. The data were expressed as means ± SEM. *P*‐value < 0.05 was considered to be statistically significant.

## 3 RESULTS

### 3.1 Clinical characteristics of TLE and control subjects

Fourteen TLE patients (eight males and six females) were selected in our research, and the mean age was 22.36 ± 2.00 years (range from 12 to 36 years) (Table 1). Meanwhile, 10 control patients (four males and six females) were enrolled, and the mean average was 21.60 ± 2.38 years (range from 11 to 34 years) (Table 2). No significant difference in age (*P* > .05) or sex (*P* > .05) was found between the TLE and control groups.

**Table 1:** Clinical characteristics of TLE patients

No.	Sex	Age (year)	Durations (years)	AEDs	Resected tissue	Pathology result
1	F	21	4	VPA,PB,CBZ,LEV	LTN	G,NL,ND
2	F	12	5	OXC,VPA,GBP	LTN	G,NL
3	M	24	5	CBZ,PHT,PB,LTG	LTN	G,NL,ND
4	M	30	7	VPA,PB,CBZ	RTN	G,NL,ND
5	M	36	18	CBZ,VPA,CLB,TPM	LTN	G,NL,ND
6	F	28	11	VPA,CBZ,PHT	RTN	G,NL
7	M	16	7	OXC,VPA,PHT	RTN	G,NL
8	M	13	7	LTG,TPM,CBZ	RTN	G
9	M	20	8	CBZ,PB,LTG,LEV	RTN	G,NL
10	F	25	8	CBZ,PB,LTG,LEV	RTN	G,NL
11	M	15	9	LTG,TPM,CBZ	RTN	G,NL
12	F	17	5	VPA,CBZ,TPM	RTN	G,NL,ND
13	F	23	8	OXC,VPA,TPM	RTN	G,NL
14	M	33	12	CBZ,PHT,LTG	RTN	G,NL,ND

Abbreviations: AEDs, antiepileptic drugs; CBZ, carbamazepine; CLB, clonazepam; GBP, gabapentin; LEV, levetiracetam; LTG, lamotrigine; M, male; ND, neuronal degeneration; NL, neuronal loss; OXC, oxcarbazepine; Pathology: G, gliosis; PB, phenobarbitone; PHT, phenytoin; Resected tissue: LTN, left temporal neocortex; RTN, right temporal neocortex; Sex: F, female; TPM, topiramate; VPA, valproic acid.

**Table 2:** Clinical characteristics of control patients

No.	Sex	Age (year)	Etiology diagnosis	Resected tissue	Seizure	Pathology result
1	F	11	Brain trauma	LTN	None	Normal
2	F	27	Brain trauma	LTN	None	Normal
3	F	13	Brain trauma	LTN	None	Normal
4	F	31	Brain trauma	RTN	None	Normal
5	F	22	Brain trauma	RTN	None	Normal
6	M	16	Brain trauma	RTN	None	Normal
7	M	24	Brain trauma	LTN	None	Normal
8	M	18	Brain trauma	LTN	None	Normal
9	M	20	Brain trauma	RTN	None	Normal
10	F	34	Brain trauma	LTN	None	Normal

Abbreviations: LTN, left temporal neocortex; M, male; RTN, right temporal neocortex; Sex: F, female.

### 3.2 Localization of STK24 in TLE patients and PTZ‐kindled mice

**FIGURE 1:**
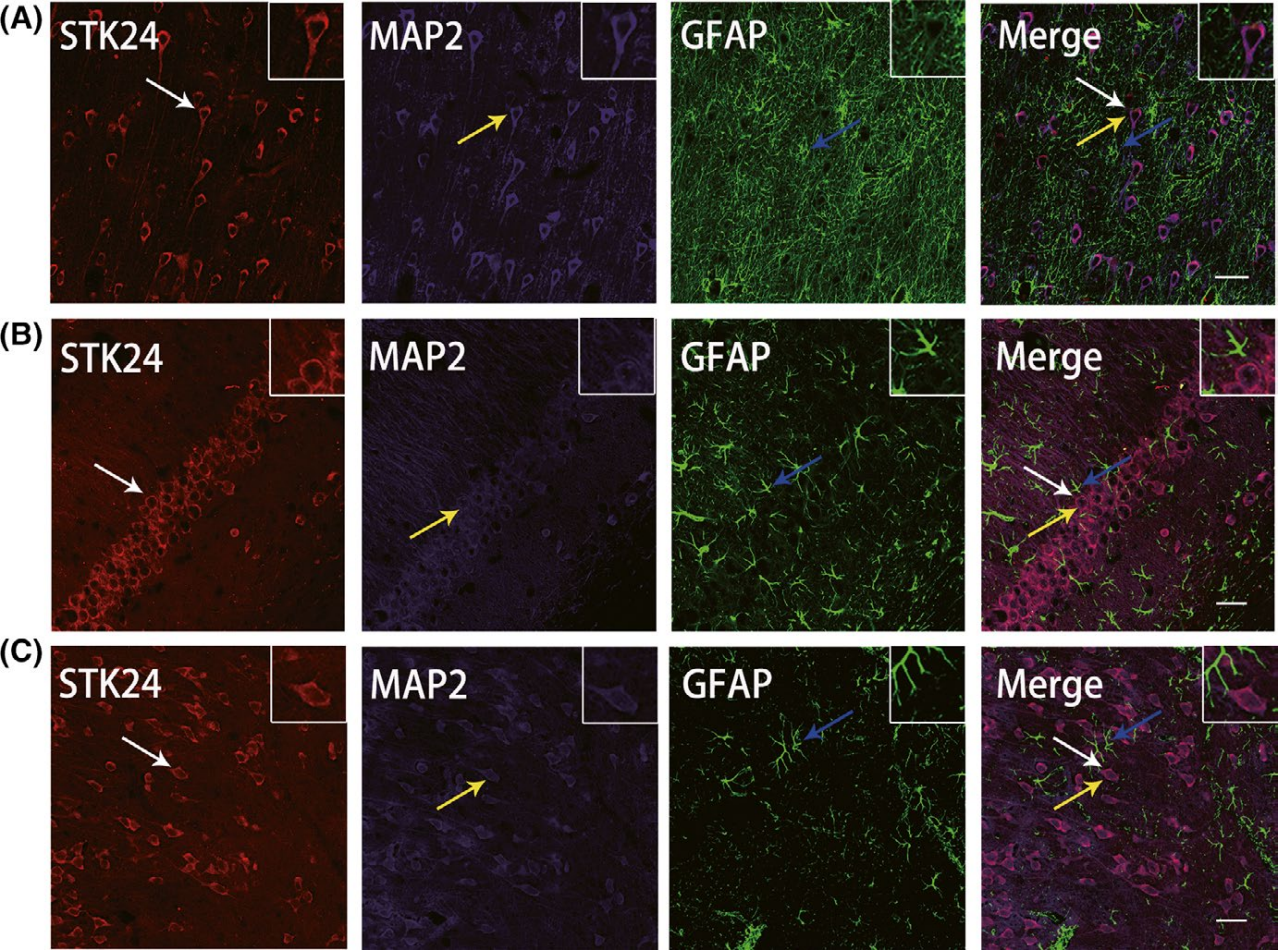
Localization of STK24 in epileptic patients and PTZ‐kindled mice. A, The localization of STK24‐positive cells in the anterior temporal cortex from TLE patients by immunofluorescence staining: STK24 (red) and MAP‐2 (purple), but not GFAP (green), coexpressed (merged) in the anterior temporal cortex of TLE patients. B, C, The localization of STK24‐positive cells in the hippocampal CA1 region and temporal cortex of PTZ‐kindled mice. Also, STK24 (red) was colocalized with MAP2‐positive neurons (purple) but did not with GFAP‐positive astrocytes (green) in the hippocampal CA1 region (B) and the anterior temporal cortex (C). White arrows: STK24‐positive cells, yellow arrows: MAP2; blue arrows: GFAP‐positive cells (scale bar = 100 μm)

Immunofluorescence labeling was performed as previously described. In the anterior temporal cortex from TLE patients, STK24 (red) was mainly located in the cytoplasm of the neuron and coexpressed with the neuronal marker MAP2 (purple), but not with the astrocyte marker GFAP (green) (Figure 1A). At the same time, STK24 was also observed in the cytoplasm of the neuron but not in the astrocyte in the hippocampus (Figure 1B) and anterior temporal cortex (Figure 1C) of PTZ‐kindled mice. In summary, consistent with earlier reports, STK24 is still located in neurons specifically and mainly presented in the cytoplasm of the neuron.

### 3.3 Decreased STK24 expression in TLE patients and PTZ‐kindled mice

**FIGURE 2:**
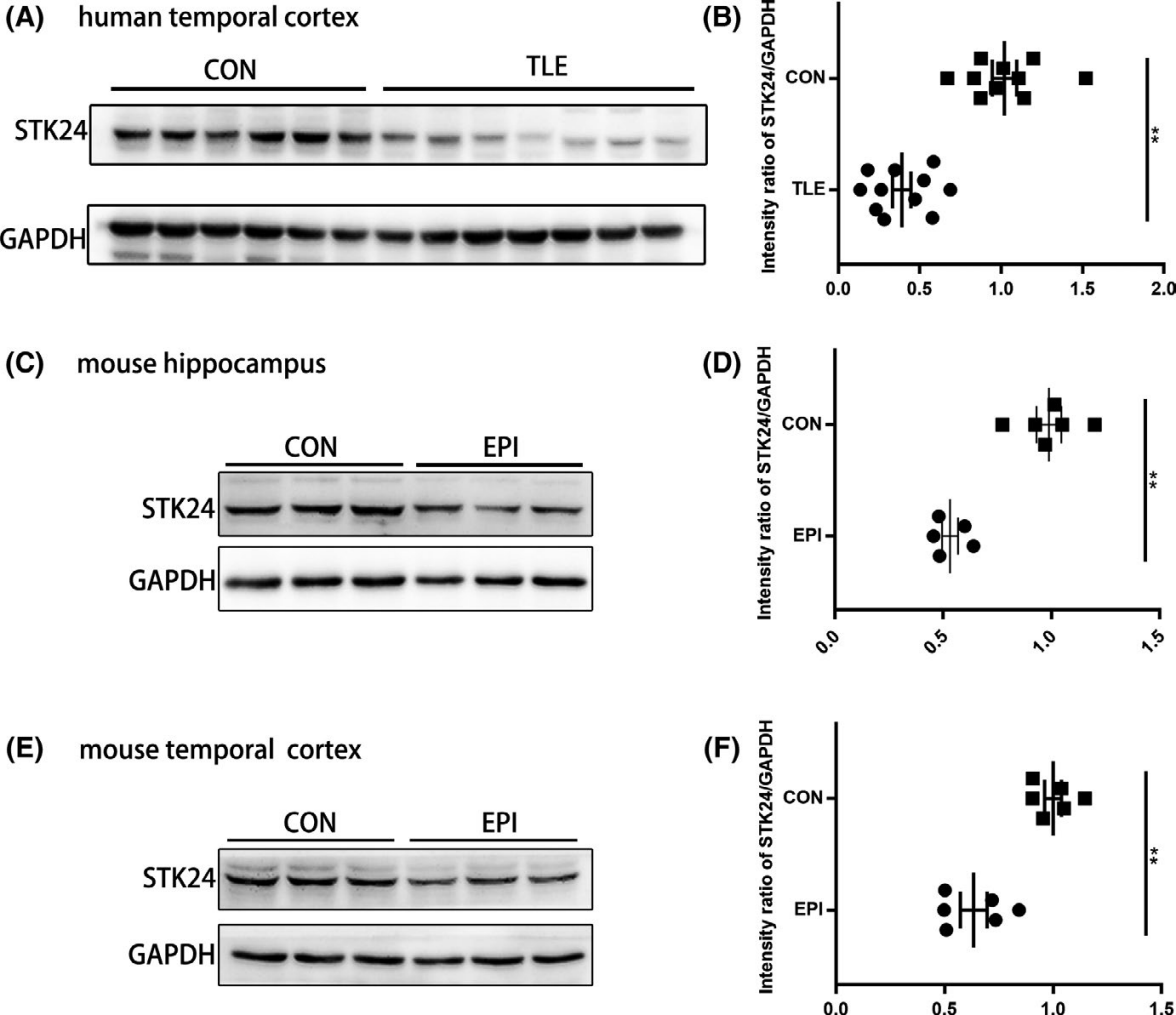
Decreased STK24 expression in epileptic patients and PTZ‐kindled mice (A) Representative images of STK24 expression in the anterior temporal cortex of human. B, Comparison of the Western blot intensity ratios of STK24/GAPDH in the anterior temporal cortex of human (n = 10 in the control group and n = 11 in TLE group, technical replicates four times, **P < .01). C, E, Representative Western blot images of STK24 expression in the mouse hippocampus (C) and anterior temporal cortex (E). D, F, Statistical graphs show the comparison of the Western blot intensity ratio of STK24/GAPDH in the mouse hippocampus (D) (n = 6 in the control group and n = 5 in the epileptic group, technical replicates four times, an unqualified hippocampal sample which was removed from the epileptic group were shown in Figure S2, **P < .01) and anterior temporal cortex (F) (n = 6 in the control group and n = 6 in the epileptic group, technical replicates four times, **P < .01)

STK24 expression in the anterior temporal cortex from TLE patients and control patients was detected by Western blot. As shown in Figure 2A and B, STK24 expression was significantly decreased in TLE patients compared with that in control patients (mean intensity ratio: 0.39 ± 0.06 in TLE patients and 1.02 ± 0.07 in control patients) (**P < .01). Simultaneously, Western blot was also performed to investigate the STK24 expression in PTZ‐kindled mice and revealed a similar decrease in the hippocampus and anterior temporal cortex in PTZ‐kindled mice (mean intensity ratio: 0.53 ± 0.04 in the hippocampus and 0.63 ± 0.06 in the anterior temporal cortex) compared with that in control mice (0.99 ± 0.06 in the hippocampus and 1.00 ± 0.04 in the anterior temporal cortex) (**P < .01) (Figure 2C–F).

### 3.4 SREDs induces a significant decrease of STK24 expression

**FIGURE 3:**
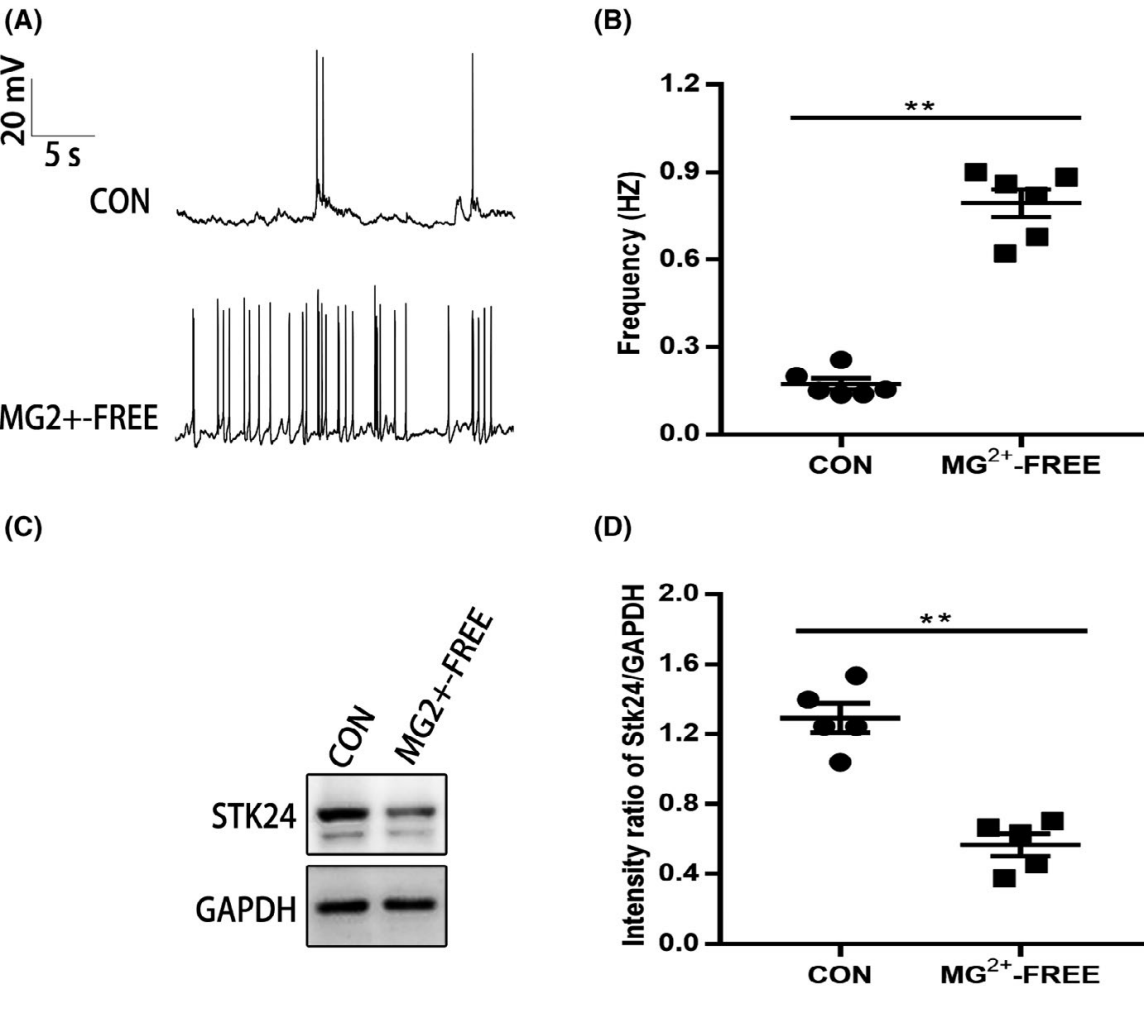
SREDs induce the decrease of STK24 expression. A, Representative trace of AP in the Mg^2+^‐free group and the control group. B, Comparison of the AP frequency in two groups (three independent primary cultures with six neurons; **P < .01). C, Representative images of STK24 expression in the SREDs. D, The statistical graph shows the comparison of the Western blot intensity ratio of STK24/GAPDH in two groups (five independent primary cultures, technical repeated 3 times; **P < .01)

Owing to complex and compensatory environmental changes in regulating metabolic homeostasis of the living animal, it is difficult to evaluate the electrophysiological function at the single neuron and molecular levels in vivo studies.^20^ Hence, in our present study, a SREDs cell model of cultured hippocampal neurons was set up as previously reported,^17^, ^21^ and the whole‐cell recordings were conducted to evaluate the level of the electrical activity of cultured neurons. The results show that the frequency of action potential (AP) in the Mg^2+^‐free group was significantly higher when compared with the control group (Figure 3A and B) (control: 0.17 ± 0.02, Mg^2+^‐free: 0.79 ± 0.05) (**P < .01), and this confirms that the model was successfully established. Furthermore, Western blot was performed to detect the STK24 expression, and a significantly decreased level of STK24 was detected in the Mg^2+^‐free group compared with the control group (Figure 3C and D) (control: 1.29 ± 0.08, Mg^2+^‐free: 0.57 ± 0.06, five independent primary cultures, technical repeated three times) (**P < .01).

### 3.5 STK24 affects the excitatory synaptic transmission

**FIGURE 4:**
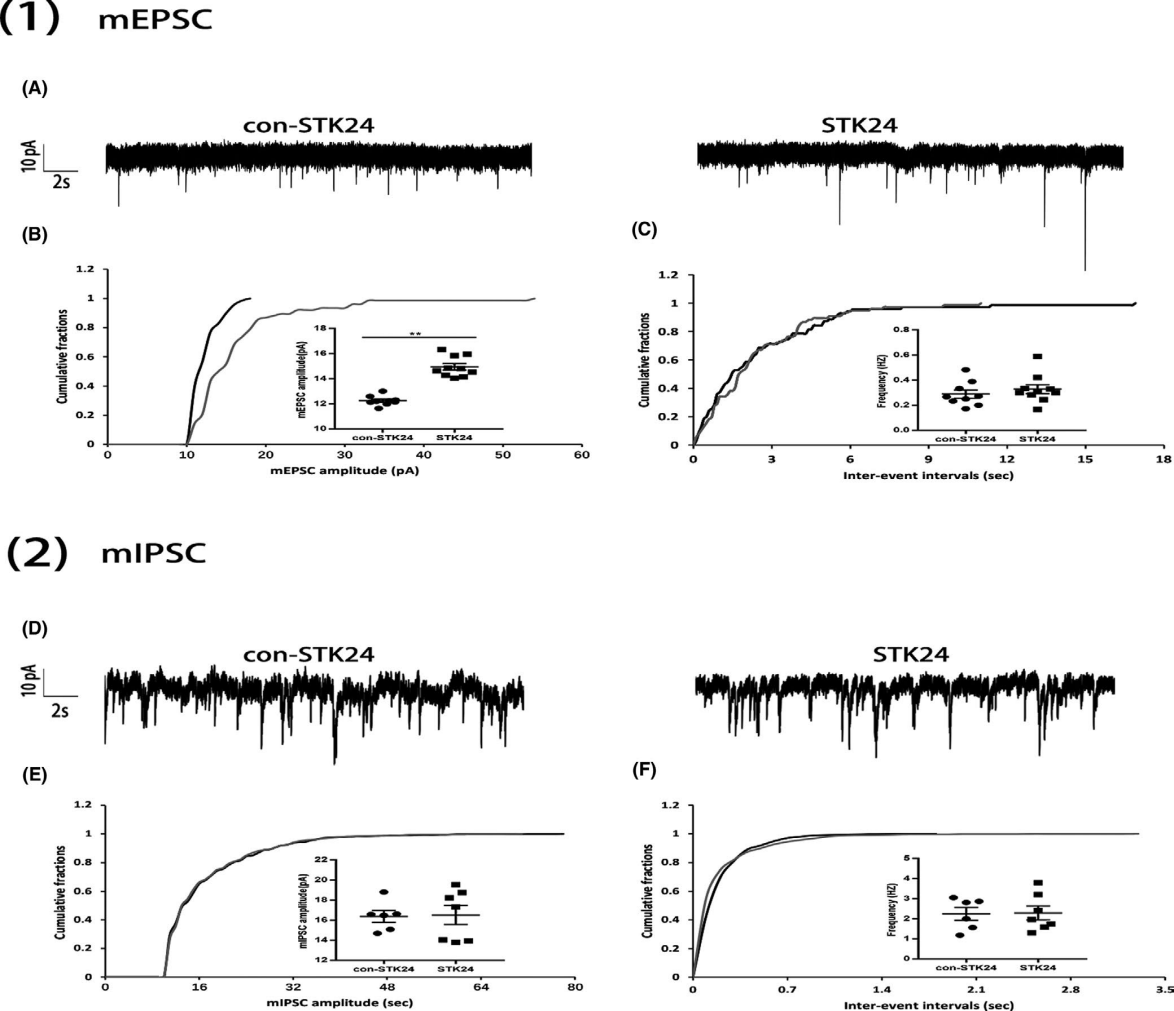
STK24 affects excitatory synaptic transmission. A, Representative traces of mEPSCs in the control group (left) and STK24 overexpressed group (right) in the Mg^2+^‐free medium. B, C, STK24 overexpression significantly increased the average mEPSCs amplitude (B), but not influence the average mEPSCs frequency (C), compared with the control group (control group: n = 9, STK24 overexpressed group: n = 10, four independent primary cultures, **P < .01). D, Representative traces of mIPSCs in the control group (left) and STK24 overexpressed group (right) in the Mg^2+^‐free medium. E, F, STK24 overexpression does not influence the average mIPSCs amplitude (E) and frequency (F), compared with the control group (control group: n = 6, STK24 overexpressed group: n = 7, four independent primary cultures, *P* > .05)

To examine the role of STK24 on neuronal excitability in epilepsy, we measured miniature excitatory and inhibitory postsynaptic currents (mEPSCs and mIPSCs) of STK24 overexpressed cultured hippocampal neurons and corresponding control neurons in Mg^2+^‐free medium via whole‐cell recordings. Representative traces of mEPSCs and mIPSCs were, respectively, shown in Figure A and D. In the STK24 overexpressed group, the cumulative distribution of mEPSCs amplitudes demonstrated a right shift and the mean mEPSCs amplitudes were increased when compared with the control group (Figure 4B) (control: 12.25 ± 0.13, STK24: 14.94 ± 0.26) (**P < .01). Meanwhile, no significant difference in the cumulative distribution of mEPSCs frequency and mean mEPSCs frequency was found between the STK24 overexpressed group and the control group (Figure 4C) (control: 0.29 ± 0.03, STK24: 0.33 ± 0.04) (*P* > .05). At the same time, no significant difference was discovered in the amplitude or frequency of mIPSCs between two groups (Figure 4E and F) (control: 16.38 ± 0.59 in amplitude, 2.25 ± 0.32 in frequency; STK24: 16.52 ± 0.95 in amplitude, 2.29 ± 0.35 in frequency) (*P* > .05). Thus, STK24 overexpression elevated the neuronal excitability, presumably via influencing the function of excitatory synaptic transmission. Furthermore, this specific change in amplitude of mEPSCs largely suggests that STK24 overexpression may affect on the excitatory postsynaptic element.

### 3.6 STK24 is a potential binding partner of the NMDA receptor complex

**FIGURE 5:**
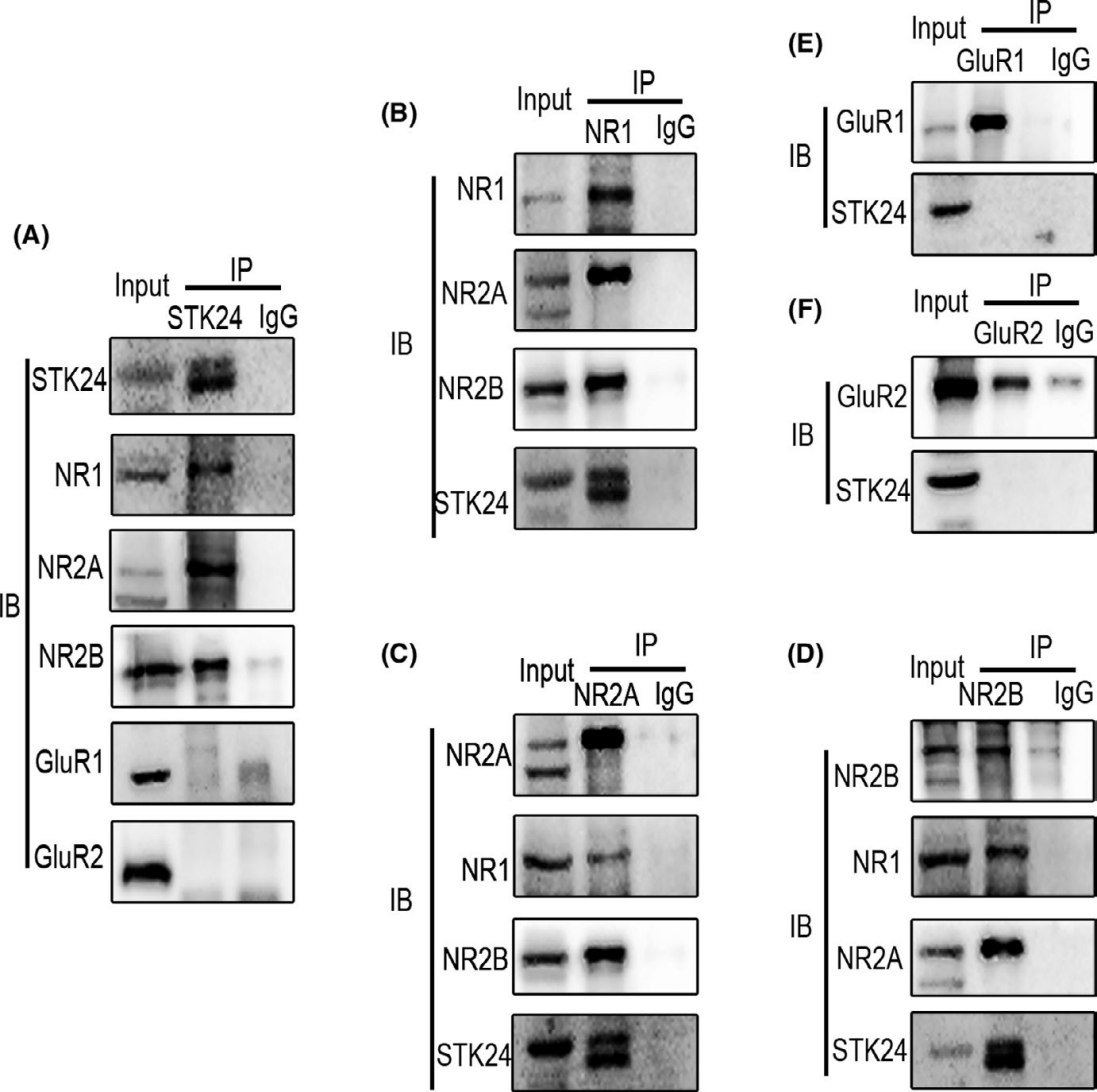
STK24 is a potential binding partner of the NMDAR complex in the hippocampus of PTZ‐kindled mice. A, STK24 interacts with the NMDAR complex, but not with the AMPAR complex. Hippocampal lysates from PTZ‐kindled mice were immunoprecipitated with STK24 antibody and incubated with corresponding antibodies (IB: immunoblotting, IP: immunoprecipitation). B–F, Lysates from hippocampus of PTZ‐kindled mice were immunoprecipitated with antibodies against NR1(B), NR2A(C), NR2B(D), GluR1(E), GluR2(F), and incubated with corresponding antibodies

**FIGURE 6:**
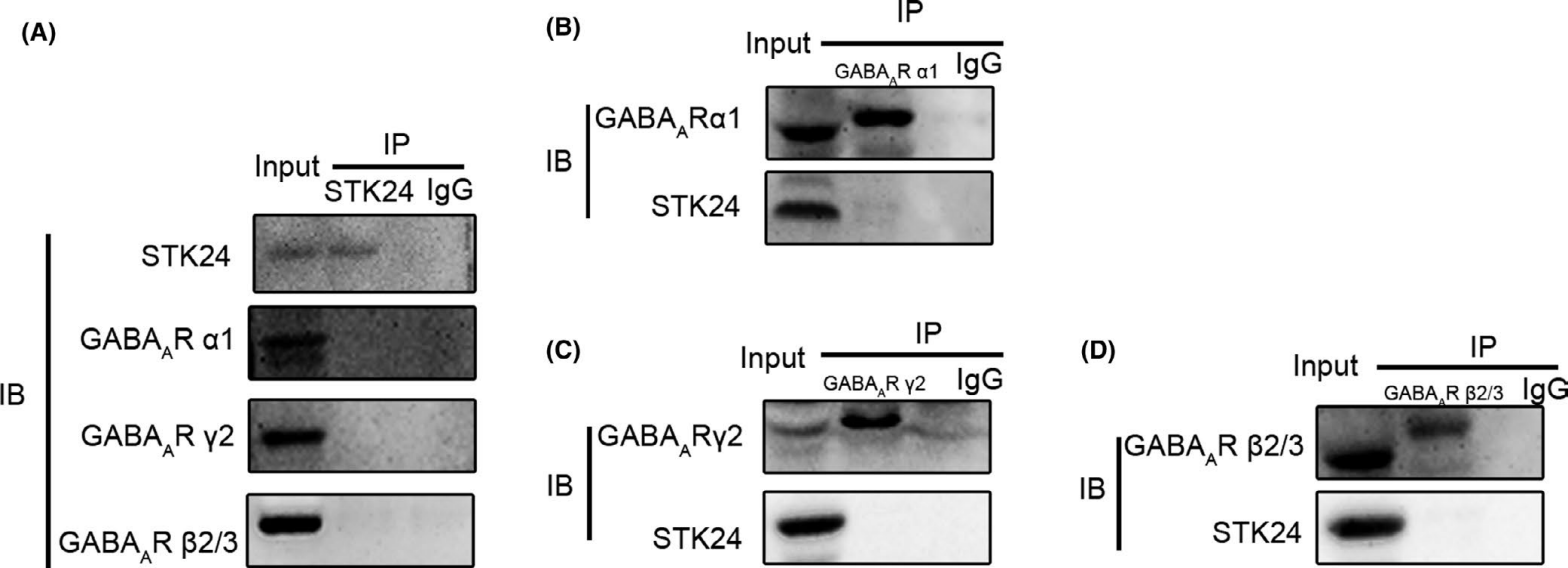
STK24 is not a binding partner of the GABA_A_R complex in the hippocampus of PTZ‐kindled mice. A, STK24 is not within the GABA_A_R complex. Extracts from hippocampal lysates of PTZ‐kindled mice were immunoprecipitated with antibodies against STK24 and blotted with corresponding antibodies. B–D, Extracts from hippocampal lysates of PTZ‐kindled mice were immunoprecipitated with antibodies against GABA_A_R α1(B), GABA_A_R γ2(C), GABA_A_R β2/3(D), and blotted with corresponding antibodies

Previous research reported that STK24 promotes the maturation of excitatory dendritic spines, which indicate changes in functional synapses.^8^ Besides, the excitatory glutamatergic receptors, mainly including AMPAR and NMDAR, are major receptors which could influence the excitatory synaptic transmission.^22^ Given the association of glutamate receptors and excitatory synaptic transmission, we suspected that STK24 may interact with some subunits of excitatory glutamate receptors. However, we did not know whether this effect was caused by NMDAR or AMPAR. To investigate the relationship between STK24 and excitatory postsynaptic receptors, coimmunoprecipitation was performed to detect the association between endogenous STK24 and main subunits of NMDARs and AMPARs in hippocampal lysates from PTZ‐kindled mice. These results suggested some possible interactions between STK24 and NR1, NR2A and NR2B (Figure 5A, B‐D), but no interaction between STK24 and GluR1, GluR2 (Figure 5A, E‐F), which indicates that STK24 is a potential binding partner of the NMDA receptor complex. We also detected the interaction between STK24 and main subunits of GABA_A_R to find out whether STK24 affects inhibitory postsynaptic receptors, while the results did not show any interaction between STK24 and GABA_A_R α1, GABA_A_R γ2, or GABA_A_R β2/3 (Figure 6A‐D). In a word, these results indicated that STK24 might specifically interact with the NMDAR complex in vivo.

## 4 DISCUSSION

Excitatory synaptic transmission, which is modulated mainly by the synaptic vesicle formation, release, and recycling, postsynaptic receptors, and their regulators,^23^ plays a critical role in many neurological diseases, including epilepsy.^23^, ^24^ Regulating excitatory synaptic transmission is the key step to control the development of epilepsy.^23^, ^24^, ^25^ STK24 has multiple functions in synaptic activity, including promoting dendritic spine and excitatory synapse development^6^, ^8^, ^9^, ^26^, ^27^, ^28^ and changing neuronal excitability.^9^ According to the fundamental characteristics of STK24, we hypothesize that STK24 may involve in epilepsy.

STK24 mRNA was found widely expressed in different rat brain regions and especially abundantly expressed in the cerebral cortex and hippocampus.^29^ Besides, STK24 was identified expressed in neurons of the adult rat hippocampus and the neuronal layers of the occipital cortex.^6^ For now, our study demonstrated that STK24 mainly expressed in the neuronal cytoplasm rather than astrocytes in the temporal cortex of TLE patients and the temporal cortex and hippocampus of PTZ‐kindled mice. These results suggested that STK24 specifically located in epileptic neurons, which indicates that STK24 probably has some effects on neurons in epilepsy. Subsequently, the Western blot indicated that the expression level of STK24 was significantly down‐regulated in TLE patients and PTZ‐kindled mice. These results indicate that STK24 may have a potential regulatory effect on epilepsy.

In order to figure out whether STK24 has a regulatory effect on epilepsy, the whole‐cell recordings were performed to detect the synaptic transmission of STK24 overexpressed neurons in Mg^2+^‐free medium, which can simulate spontaneous recurrent epileptiform discharges in epilepsy.^30^ The cell model was chosen to rule out some complex extra‐synaptic factors and keep all the external conditions consistent, so that a better observation of the intrinsic electrophysiological activity of a single synapse can be conducted. In SREDs cell model, overexpression of STK24 altered mEPSCs, but not mIPSCs, suggesting the excitatory synaptic transmission of the epileptic hippocampal neuron was increased. Meanwhile, overexpressing STK24 resulted in the change of the amplitude other than the frequency of mEPSCs indicated that STK24 could affect on the excitatory postsynaptic element.^31^, ^32^, ^33^, ^34^

In order to provide further evidence to support the regulation of excitatory synaptic transmission by STK24, possible interactions between STK24 and main glutamate and GABA receptors were detected by coimmunoprecipitation in the hippocampus of PTZ‐kindled mice in this study. Be consistent with what we have expected, the results suggested that STK24 particularly interacted with main subunits of NMDARs, including NR1, NR2A, and NR2B. At present, extensive pieces of literature have demonstrated that the dynamic of NMDARs is indispensable for excitatory synaptic transmission.^35^ In TLE patients, NMDARS were mainly increased in dentate granule cells^36^, ^37^; meanwhile, in epileptic animal models, the increase of NMDARs could also be detected.^38^, ^39^ So far, massive efforts in basic and clinical research of NMDARs have been made during the past three decades, and they are emerging as promising new targets for epilepsy therapy.^40^, ^41^, ^42^ Therefore, STK24 overexpression may promote excitatory synaptic transmission in epileptic neurons through a certain interaction with the NMDAR complex.

In conclusion, the experimental evidence suggests that the expression level of STK24 was decreased in epileptic patients, PTZ‐kindled mice, and SRED cell model, and overexpression of STK24 in SRED cell model increased the excitatory postsynaptic transmission. All these evidence give us a hint that the decreased expression of STK24 may have an inhibitory effect on epileptogenesis via decreasing the excitatory postsynaptic transmission. However, this is a hypothesis, and further study will be needed to find out whether intervene the expression of STK24 could affect epileptogenesis. Meanwhile, although we have preliminarily confirmed the possible interactions between STK24 and NMDARs, further elucidation of the detailed mechanism of STK24 on the NMDAR is still needed.

## CONFLICTS OF INTEREST

There is no conflict of interest.

## Supporting information

Figure S1

Figure S2
