# Supplementary material for: STK24 modulates excitatory synaptic transmission in epileptic hippocampal neurons
Source: CNS Neurosci Ther. 2020 May 21;26(8):851–61. doi: 10.1111/cns.13391 (PMC7366740; doi:10.1111/cns.13391)

Full unedited blots for FIGURE 2A

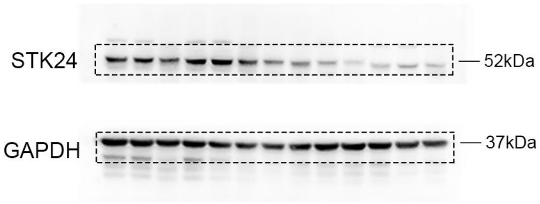

Full unedited blots for FIGURE 2C

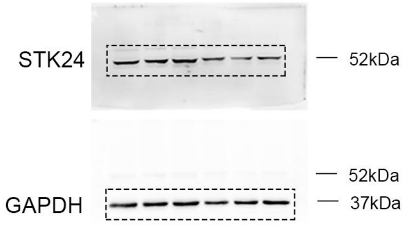

Full unedited blots for FIGURE 2E

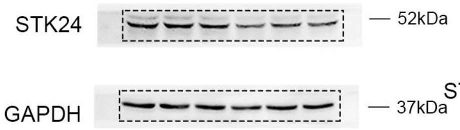

Full unedited blots for FIGURE 3C

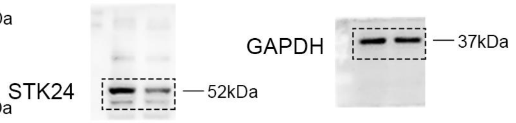

Full unedited blots for FIGURE 5A

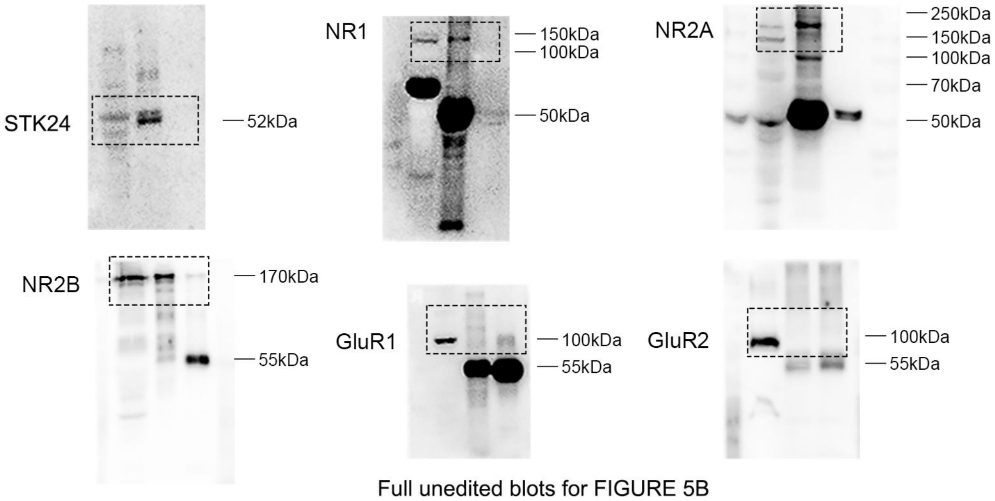

Full unedited blots for FIGURE 5B

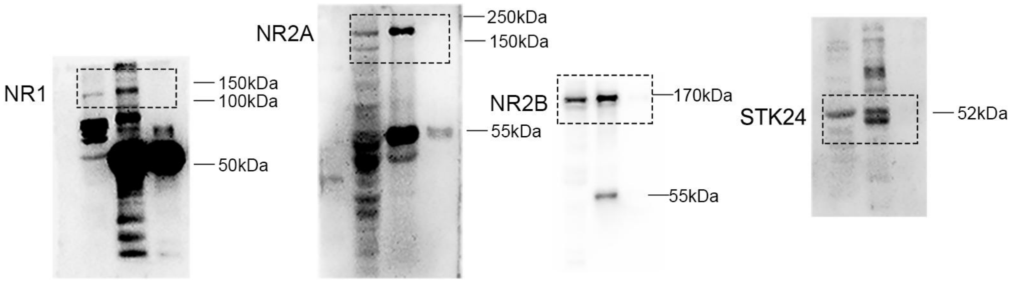

Full unedited blots for FIGURE 5C

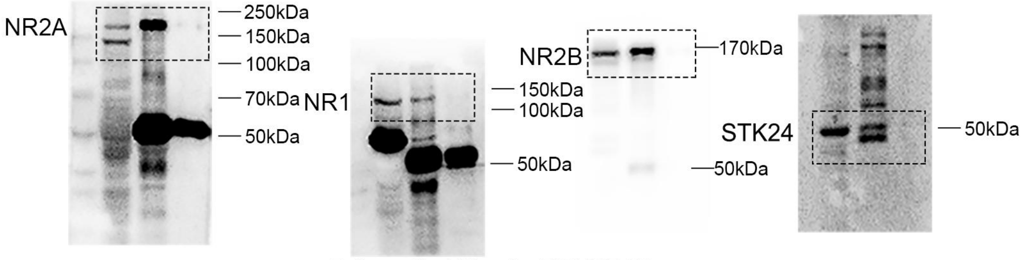

Full unedited blots for FIGURE 5D

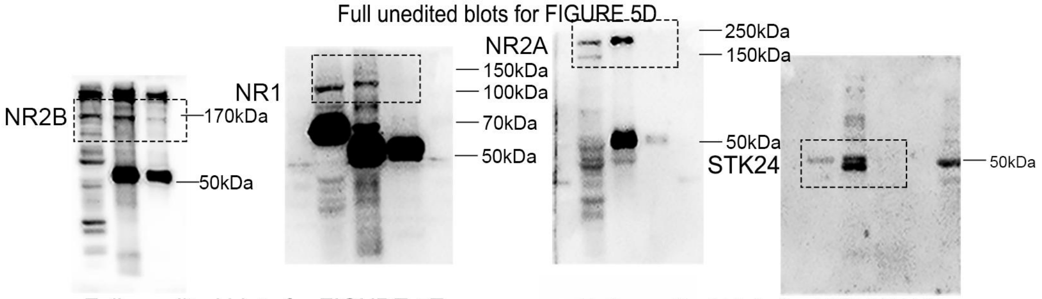

Full unedited blots for FIGURE 5E

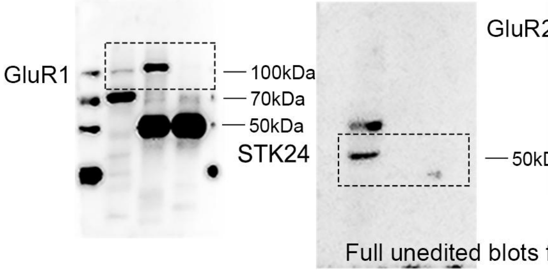

Full unedited blots for FIGURE 5F

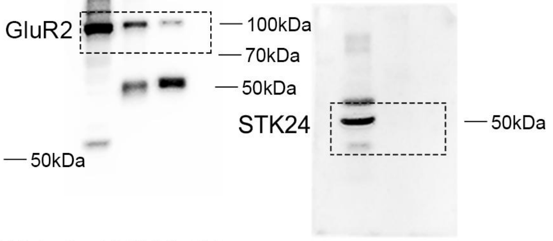

Full unedited blots for FIGURE 6A

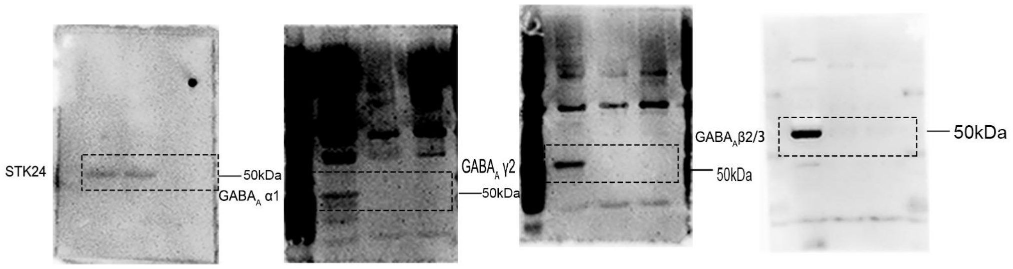

Full unedited blots for FIGURE 6B

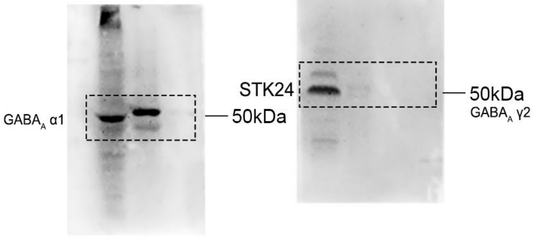

Full unedited blots for FIGURE 6C

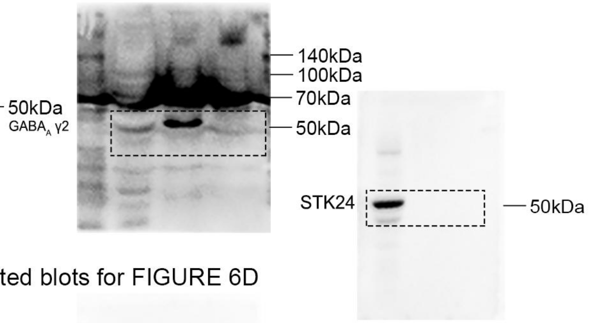

Full unedited blots for FIGURE 6D

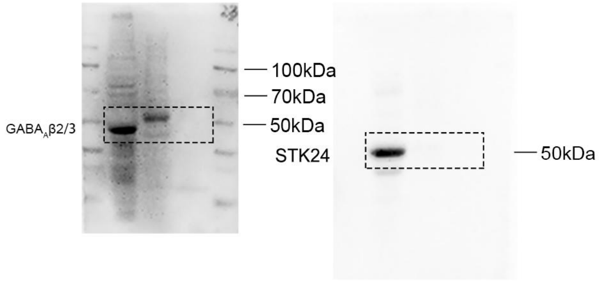

Supplement: Supplementary file 1 — Figure S1 [file CNS-26-851-s001.pdf]
