# Supplementary figures and images for: STK24 modulates excitatory synaptic transmission in epileptic hippocampal neurons
Source: CNS Neurosci Ther. 2020 May 21;26(8):851–61. doi: 10.1111/cns.13391 (PMC7366740; doi:10.1111/cns.13391)

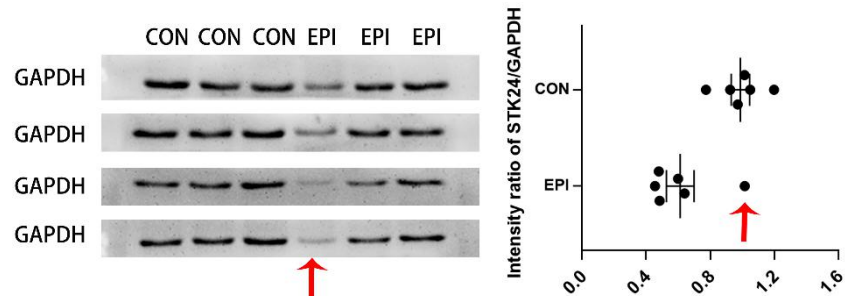

Supplement: Supplementary file 2 — Figure S2 [file CNS-26-851-s002.pdf]
